# High JAK2 Protein Expression Predicts a Poor Prognosis in Patients with Resectable Pancreatic Ductal Adenocarcinoma

**DOI:** 10.1155/2020/7656031

**Published:** 2020-09-21

**Authors:** Yang Song, Mei-Yue Tang, Wei Chen, Zhe Wang, Si-Liang Wang

**Affiliations:** ^1^Department of Oncology, Shengjing Hospital of China Medical University, 36 Sanhao Road, Shenyang 110004, China; ^2^Department of Pathology, Shengjing Hospital of China Medical University, 36 Sanhao Road, Shenyang 110004, China

## Abstract

**Background:**

Pancreatic ductal adenocarcinoma (PDAC) is one of the most fatal malignancies worldwide. The JAK/STAT signaling pathway is involved in pancreatic cancer tumorigenesis. However, the prognostic value of JAK2 expression in resectable PDAC is unclear.

**Method:**

In this study, we performed a clinicopathological analysis of 62 resectable PDAC cases with a primary focus on survival. JAK2 expression was examined by immunohistochemistry. The relationship between JAK2 expression and clinicopathological features and prognosis was analyzed.

**Results:**

Survival curve analyses revealed that high levels of JAK2 expression predict a poor prognosis in resectable PDAC patients. Multivariate analysis confirmed that JAK2 expression can predict the prognosis of PDAC.

**Conclusions:**

Assessment of JAK2 protein expression may be a promising method to predict prognosis in patients with resectable PDAC.

## 1. Introduction

Pancreatic ductal adenocarcinoma (PDAC) ranks fourth as the cause of tumor-related death and has 1-year and 5-year survival rates of 25% and <5%, respectively [1]. Compared with other cancer types, in which there has been a steady improvement in survival, little progress has been made in pancreatic cancer. Because the pancreas is retroperitoneal, tumor-associated symptoms do not appear until the tumor progresses to an advanced stage or until the development of distant metastasis, when surgical treatment is not indicated [2]. FOLFIRINOX- (5-FU, leucovorin, irinotecan, and oxaliplatin) based chemotherapy with fractionated external beam radiotherapy is the standard treatment for unresectable pancreatic cancer patients [3]. The combination of gemcitabine plus albumin-bound paclitaxel is a preferred option for metastatic patients [4]. However, the prognosis of these patients remains poor. To date, immune checkpoint inhibitors have not shown significant positive results in the treatment of pancreatic cancer. The use of exosome-based immunotherapies for PDAC treatment was recently proposed [5]. However, the biological behavior of tumor cells determines the rate of early recurrence and metastasis and the chemo- and radioresistance of pancreatic cancer [6].

Various biomarkers, such as serum carbohydrate antigen 19-9 (CA19-9) levels, noncoding RNAs [7], programmed cell death 1/programmed cell death ligand 1 [8], and circulating tumor DNA [9], can predict the prognosis of PDAC. However, identifying a prognostic marker involved in the progression of pancreatic cancer would be valuable [10]. The Janus kinase/signal transducer and activator of transcription (JAK/STAT) signaling pathway plays a role in pancreatic cancer [11, 12] and is responsible for tumor initiation and progression [13]. JAK/STAT pathway inhibition is a novel treatment strategy targeting the proliferation and survival of pancreatic cancer cells [14] and the catabolic response to malignancy [15]. A previous study [16] showed that the JAK1/JAK2 inhibitor ruxolitinib has potential clinical benefit. However, whether JAK2 is a prognostic marker for PDAC remains unknown.

The aim of this study was to explore the prognostic value of JAK2 expression in patients with resectable pancreatic cancer. Immunohistochemistry was used to evaluate JAK2 expression in human pancreatic cancer tissues, and its relationship with the clinical outcome was estimated. The findings of this study may improve our knowledge of the clinical significance and prognostic value of JAK2 expression in pancreatic cancer patients.

## 2. Materials and Methods

### 2.1. Patient Characteristics

The medical records of patients with resectable pancreatic carcinoma treated between January 2010 and December 2016 at Shengjing Hospital of China Medical University were retrospectively reviewed. The inclusion criteria were as follows: histopathologically confirmed PDAC postsurgery, no treatments prior to surgery, the absence of other malignant tumors, and complete clinicopathological data. Sixty-two patients with PDAC were enrolled in the study.

### 2.2. Bioinformatics Analysis

A database (https://ualcan.path.uab.edu/index.html), which is based on The Cancer Genome Atlas (TCGA) database, was used to evaluate the expression pattern of JAK2 in a large number of PDAC tissues. “JAK2” and “pancreatic ductal adenocarcinoma” were used as the filter parameters.

### 2.3. Immunohistochemistry

All PDAC tissue specimens were processed in the Department of Pathology at Shengjing Hospital (Shenyang, China). Samples were formalin-fixed and paraffin-embedded and cut and mounted on slides. The JAK2 primary antibody (catalog no. BA3398, Boster Biological Technology, Wuhan, China) was diluted to 1 : 100. Slides were incubated with a primary antibody in a working solution of 100 *μ*L. Two experienced pathologists who had no access to the clinical data analyzed the staining results, which were quantified using a staining index (values, 0-12) determined by multiplying the score of staining intensity by the score of the positive area. The intensity was scored as follows: 0, negative; 1, weak; 2, moderate; and 3, strong. The frequency of positive cells was defined as follows: 0, less than 5%; 1, 5% to 25%; 2, 26% to 50%; 3, 51% to 75%; and 4, greater than 75%. JAK2 staining was classified as low expression (0 to 7) or high expression (8 to 12).

### 2.4. Statistical Analysis

Quantitative data were expressed as averages and ranges, and categorical findings were expressed as numbers and percentages. The *t*-test was adopted to compare the differences between the subgroups if the quantitative data was normally distributed; otherwise, the Mann-Whitney *U* test was used. Overall survival (OS) was defined as the period from the day of the surgery to either the last follow-up visit or the date of death. The log-rank test and Kaplan-Meier method were used for survival analysis, and the Cox regression model was used to explore the prognostic factors. *P* < 0.05 was considered statistically significant. SPSS software version 19.0 (SPSS Inc., Chicago, IL, USA) was used to analyze the data.

## 3. Results

### 3.1. Patient Information

The demographic and clinicopathological data of the 62 patients are described in Table 1. None of the patients dropped out from the follow-up, and by the end of the study, 35 patients (56.5%) died. The median age was 59 years (range, 38–78 years). There were 38 men (61.3%) and 24 women (38.7%). The median of the tumor diameter (*P*_25_-*P*_75_) is 4.28 cm. According to the eighth edition of the pancreatic cancer AJCC staging system, 4 cm is used as the threshold for T2/T3 staging. It has been shown that 4 cm is the threshold in other studies of pancreatic cancer prognosis [17, 18]. Therefore, in our study, 4 cm was selected as the cut-off of the tumor diameter. Patients in both groups accounted for half. Fifty (80.6%) patients had moderately or well-differentiated tumors, whereas 12 (19.4%) patients had poorly differentiated tumors, and 27 (43.5%) patients had lymph node metastasis. CA19-9 serum level was >37 U/mL in 49 (72.0%) patients. The median OS was 17.9 months.

### 3.2. JAK2 Expression in Resectable PDAC Tissue Samples

**Figure 1:**
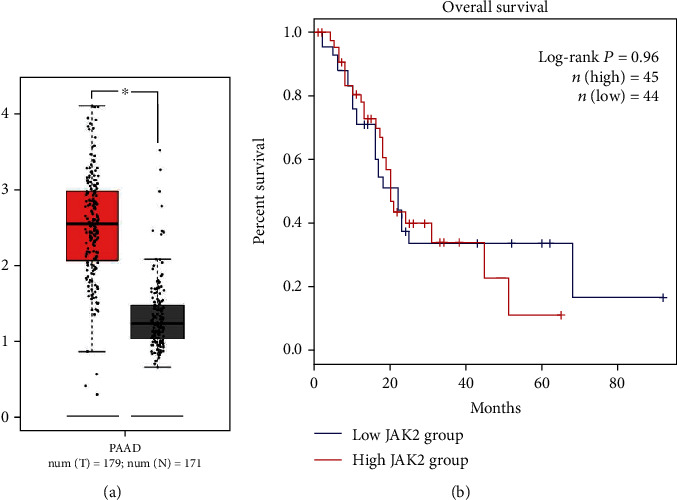
Using TCGA data to show (a) JAK2 mRNA expression and (b) the relationship between JAK2 mRNA and survival in PDAC tissues.

**Figure 2:**
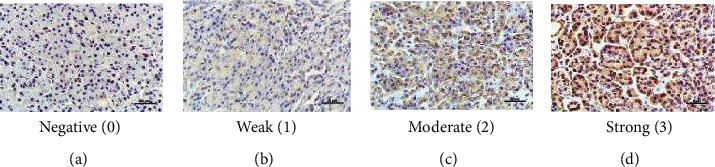
Intensity of JAK2 expression in PDAC tissue by immunohistochemistry. Positive expression is shown by brown-yellow particles distributed in the cell membrane and cytoplasm (SP staining ×50). The cellular staining was classified using a scale of 0-3 as follows: (a) 0 = negative, (b) 1 = weakly positive, (c) 2 = moderately positive, and (d) 3 = strongly positive.

JAK2 expression was analyzed by bioinformatics analysis, and the results are shown in Figure 1. According to TCGA samples, JAK2 expression was significantly elevated in PDAC tissues (*P* < 0.05). JAK2 expression was examined by immunohistochemistry in tumor tissues from 62 patients with resectable PDAC for further verification. JAK2-positive staining was defined as the distribution of brown-yellow particles in PDAC cells, as shown in Figure 2. A JAK2 staining intensity of 0 or 1 was observed in 27 (43.5%) patients, whereas a staining intensity of 2 or 3 was observed in 35 (56.5%) patients. Eight of the 12 (67%) patients with poor tumor differentiation and 27/50 (54%) of patients with well- or moderately differentiated tumors had a staining intensity of 2 or 3.

### 3.3. Analysis of Overall Survival

**Figure 3:**
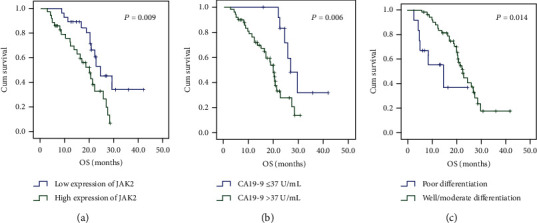
Overall survival (OS) in patients stratified by prognostic factors found to be independently associated with OS. (a) CA19‐9 > 37 U/mL predicts poor prognosis in patients with resectable pancreatic ductal adenocarcinoma. *P* = 0.006. (b) Poor differentiation predicts poor prognosis in patients with resectable pancreatic ductal adenocarcinoma. *P* = 0.014. (c) High expression of JAK2 predicts poor prognosis in patients with resectable pancreatic ductal adenocarcinoma. *P* = 0.009. Significant differences in OS were revealed by the log-rank test.

Table 2 shows the log-rank test results. JAK2 expression (*P* = 0.009), serum CA19-9 levels (*P* = 0.006), and histological tumor differentiation (*P* = 0.014) were related to OS. Other characteristics did not influence OS, such as sex, age, maximum tumor diameter, location, primary tumor category (pT), and pathological regional lymph node (pN) category (*P* > 0.05). High JAK2 expression and serum CA19-9 levels ≥ 37 U/mL were significantly related to OS according to the multivariate Cox model (Figure 3). High JAK2 expression and serum CA19-9 levels ≥ 37 U/mL were related to shorter OS independently (Table 3). These data suggested that, in addition to the conventional prognostic factor serum CA19-9 level, JAK2 expression may act as a valuable biomarker to predict the prognosis of patients with resectable PDAC.

## 4. Discussion

The JAK/STAT signaling pathway is involved in many physiological processes, such as differentiation, cell growth, immune function, and hematopoiesis [19]. The JAK/STAT pathway is persistently activated in many malignant solid tumors including lung cancer [20], breast cancer [21], colorectal cancer [22], head and neck cancer [23], pancreatic cancer [24], and some hematological diseases [25]. In pancreatic cancer, abnormal activation of STAT3 is crucial for invasion and metastasis [26]. JAK2 expression is correlated with pancreatic tumor cell metastasis and invasion [27]. Moreover, the JAK/STAT pathway is involved in pancreatic tumor cell immune escape [28]. JAK2/STAT3 pathway inhibitors can prolong the survival of mice by slowing tumor growth and stromal modification, in addition to altering immune cell infiltration [29]. In a pancreatic cancer mouse model, activation of JAK2/STAT3 signaling promotes stromal formation in tumors and can lead to gemcitabine resistance [30]. These data indicated that this signaling pathway may be related to both the prognosis and treatment of cancers. To the best of our knowledge, this is the first study providing evidence that high JAK2 protein expression levels are closely related to poor prognosis in resectable PDAC patients.

In a previous study, we showed that inhibition of JAK2 at the mRNA and protein levels can hamper cell growth and promote cell apoptosis in vivo and in vitro [31]. In a recent study, our team found that JAK2 inhibition increases the radiosensitivity of pancreatic cancer cells [32]. TCGA analysis showed that JAK2 mRNA expression was different between tumor samples and normal samples, but there was no significant difference in survival data. Considering the biological aggressive nature of PDAC, choosing a prognostic marker is of great importance to help clinicians select therapies that will avoid unnecessary toxicity. We explored the correlation between JAK2 expression, clinicopathological characteristics, and PDAC patient survival in our hospital in this study. Various clinicopathological features including high serum CA19-9 levels and increased tumor size are significantly correlated with poor OS [33, 34]. Consistent with previous studies, the present results showed that tumor differentiation and serum CA19-9 levels were associated with OS. In addition, we showed that high JAK2 expression was associated with poor survival, indicating that JAK2 expression may act as a prognostic biomarker in resectable PDAC patients.

JAK2 is closely associated with not only pancreatic cancer initiation and progression but also radiosensitivity. The present findings suggested that the combination of CA19-9 level, histological tumor differentiation, and JAK expression may provide more accurate information for the prediction of prognosis in PDAC. However, this was a retrospective study, and the results need to be verified in a large cohort.

The results of bioinformatics analysis suggested that JAK2/STAT3 signaling was activated in pancreatic cancer tissues. Activated JAK2 phosphorylates STAT3, leading to its nuclear translocation and tumorigenesis [35]. A recent study showed that the natural compound B6 (3-deoxy-2*β*,16-dihydroxynagilactone E) inhibits the phosphorylation of STAT3 by inactivating and interacting with JAK2, thereby inhibiting growth and inducing apoptosis of breast cancer cells with overactivated STAT3; this provides a novel promising strategy for the treatment of cancers with JAK2/STAT3 overactivation [36]. A Japanese study found that p-STAT3 expression was correlated with OS, and the activation of the JAK2/STAT3 pathway is critical for the survival of clear-cell ovarian cancers [37]. A different study suggested that combination of JAK2 inhibitors with immune checkpoint inhibition is effective for the treatment of non-small-cell lung cancer [38]. A dual inhibitor of STAT3 pathways induces death of tumor cells [39], indicating that suppression of the JAK2/STAT3 pathway may be a promising approach for the prevention and treatment of pancreatic cancer. As a key factor in the JAK/STAT pathway, JAK2 may be useful as a biomarker for predicting the prognosis of pancreatic cancer as well as a therapeutic target for patients with resectable PDAC.

In conclusion, JAK2 expression can serve as a novel, potent biomarker in resectable PDAC, providing independent prognostic information. The prognostic value of JAK2 expression can be improved through combination with clinical information, which supports patient risk stratification and may be used for the design of different treatment strategies.

## Figures and Tables

**Table 1:** Baseline characteristics of evaluable patients (*N* = 62).

Characteristics	No. of cases	JAK expression		*P* value
		Low	High	
Gender				
Men	38 (61.3)	22	16	0.773
Women	24 (38.7)	13	11	
Age (years)				
≥60	28 (45.2)	16	12	0.921
<60	34 (54.8)	19	15	
Location				
Head	30 (48.4)	19	11	0.289
Body/tail	32 (51.6)	16	16	
Maximum tumor diameter (cm)				
<4	31 (50.0)	17	14	0.797
≥4	31 (50.0)	18	13	
CA19-9 (U/mL)				
<37	13 (21.0)	4	9	0.035
≥37	49 (72.0)	31	18	
Differentiation				
Well/moderate	50 (80.6)	27	23	0.426
Poor	12 (19.4)	8	4	
pT category				
pT1+pT2	34 (54.8)	17	17	0.258
pT3+pT4	28 (45.2)	18	10	
pN category				
pN0	35 (56.5)	22	13	0.001
pN1	27 (43.5)	6	21	
Circumferential resection margin				
R0	46 (74.2)	27	19	0.545
R1	16 (25.8)	8	8	
Neural invasion				
Yes	23 (37.1)	13	10	0.993
No	39 (62.9)	22	17	
Vascular invasion				
Yes	16 (25.8)	11	5	0.249
No	46 (74.2)	24	22	

**Table 2:** Univariate analysis of overall survival (OS) and clinicopathological characteristics in 62 patients with resectable pancreatic ductal adenocarcinoma.

Characteristics	Median OS (months) (95% CI)	Log-rank *x*^2^	*P* value
Gender		0.05	0.824
Female	20.5 (18.9-22.0)		
Male	22.4 (19.4-25.4)		
Age (years)		0.244	0.621
≥60	24.6 (18.9-30.3)		
<60	20.5 (18.6-22.4)		
Location		0.055	0.815
Head	20.5 (17.2-23.8)		
Body/tail	22.1 (19.7-24.5)		
Maximum tumor diameter (cm)		2.231	0.135
<4	24.6 (17.6-31.6)		
≥4	20.8 (17.0-24.5)		
CA19-9 (U/mL) level		7.561	0.006
<37	26.8 (22.3-31.3)		
≥37	20.2 (17.9-22.4)		
Differentiation		6.036	0.014
Well/moderate	22.8 (19.1-26.4)		
Poor	13.8 (6.97-20.6)		
pT category		0.008	0.928
pT1+pT2	21.3 (18.4-24.2)		
pT3+pT4	22.1 (18.0-26.1)		
pN category		0.973	0.324
pN0	20.5 (17.8-23.2)		
pN1	25.8 (17.7-33.9)		
JAK2 expression		6.761	0.009
Low	24.6 (17.8-31.4)		
High	20.1 (15.1-25.1)		
Circumferential resection margin			
R0	20.8 (18.3-23.3)	0.062	0.803
R1	24.6 (17.6-31.6)		
Neural invasion		1.166	0.280
Yes	20.2 (15.1-25.3)		
No	22.8 (17.5-28.1)		
Vascular invasion		3.725	0.054
Yes	20.1 (17.3-22.9)		
No	22.8 (17.3-28.3)		

The *P* value of the serum CA19-9 level, the histological differentiation, and the JAK2 expression is ≤0.05, which means that they were significantly associated with OS.

**Table 3:** Multivariate analysis of prognostic factors independently associated with overall survival (OS) in patients with resectable pancreatic ductal adenocarcinoma.

Characteristics	Hazard ratio	95% CI	*P* value
CA19-9 (U/mL)	0.298	0.116-0.769	0.012
<37			
≥37			
Histological differentiation	2.080	0.947-4.572	0.068
Well/moderate			
Poor			
Expression of JAK2	0.476	0.230-0.988	0.046
High			
Low			

## Data Availability

The Cancer Genome Atlas (TCGA) database data used to support the findings of this study are included within the article. The present survival data used to support the findings of this study are available from the corresponding author upon request.
